# GR2ST: spatial transcriptomics prediction based on graph-enhanced multimodal contrastive learning

**DOI:** 10.1093/bioinformatics/btag209

**Published:** 2026-04-26

**Authors:** Jingli Zhou, Siyuan Li, Rui Han, Xuan Wang, Yadong Wang, Junyi Li

**Affiliations:** School of Computer Science and Technology, Harbin Institute of Technology (Shenzhen), Shenzhen, Guangdong 518055, China; School of Computer Science and Technology, Harbin Institute of Technology (Shenzhen), Shenzhen, Guangdong 518055, China; School of Computer Science and Technology, Harbin Institute of Technology (Shenzhen), Shenzhen, Guangdong 518055, China; School of Computer Science and Technology, Harbin Institute of Technology (Shenzhen), Shenzhen, Guangdong 518055, China; Guangdong Provincial Key Laboratory of Novel Security Intelligence Technologies, Harbin Institute of Technology (Shenzhen), Shenzhen, Guangdong 518055, China; Center for Bioinformatics, Faculty of Computing, Harbin Institute of Technology, Harbin, Heilongjiang 150001, China; Key Laboratory of Biological Bigdata, Ministry of Education, Harbin Institute of Technology, Harbin, Heilongjiang 150001, China; School of Computer Science and Technology, Harbin Institute of Technology (Shenzhen), Shenzhen, Guangdong 518055, China; Guangdong Provincial Key Laboratory of Novel Security Intelligence Technologies, Harbin Institute of Technology (Shenzhen), Shenzhen, Guangdong 518055, China; Key Laboratory of Biological Bigdata, Ministry of Education, Harbin Institute of Technology, Harbin, Heilongjiang 150001, China

## Abstract

**Motivation:**

Spatial transcriptomics techniques capture gene expression data and spatial coordinates, while simultaneously correlating them with tissue section images. This advantage makes Spatial transcriptomics data highly valuable for research, such as investigating disease mechanisms and cancer prognosis. However, the extended time and high cost of spatial transcriptomic sequencing currently limit further advancements in this field. The development of numerous deep learning methods aimed at predicting spatial transcriptomics from histology images has advanced significantly. However, these approaches often lack the ability to effectively integrate histology images with spatial transcriptomic data. Here, we propose GR2ST, a deep learning model that learns the underlying connections between image features and gene expression to predict spatial transcriptomics.

**Results:**

GR2ST leverages a large pre-trained pathology model to extract high-level histological features. We designed a dual-branch graph architecture, consisting of a dynamic threshold-based functional graph and a radius-constrained spatial graph, to capture complex spot interactions within heterogeneous tissues. The model aligns histology images with gene expression representations through a multimodal contrastive learning framework. It achieves adaptive gene expression generation via a Cell-Type Guided Multi-Branch Regression Head supervised by a context-aware weighting network, which is further integrated with cross-sample retrieval to construct an ensemble prediction. The performance of the model is evaluated on three cancer-related spatial transcriptomics datasets, including cutaneous squamous cell carcinoma and two human breast cancer cohorts, to demonstrate its effectiveness and robustness.

**Availability:**

https://github.com/zjl1109294570/GR2ST.

## 1 Introduction

In 2020, Nature Methods named spatial transcriptomics the “Technology of the Year” (Marx 2021). Unlike conventional transcriptomic approaches, spatial transcriptomics profiles gene expression within intact tissue architecture (Alon et al. 2021, Chen et al. 2022), providing new opportunities to study cellular communication and molecular signaling (Longo et al. 2021). A growing number of computational methods have been developed to integrate spatial transcriptomics with spatial information for tasks such as spatial domain identification (Dong and Zhang 2022, Xu et al. 2024) and deconvolution (Song and Su 2021, Kleshchevnikov et al. 2022). However, current spatial transcriptomics technologies remain costly and time-consuming, limiting their broader use. By contrast, hematoxylin and eosin (H&E)-stained histology slides are inexpensive and widely available (Waylen et al. 2020). As a result, predicting spatial transcriptomics from histology images has emerged as an efficient alternative.

Several methods have been proposed to predict spatial gene expression from histology images. ST-net (He et al. 2020) uses DenseNet (Huang et al. 2017)  to extract patch-level visual features and project them into transcriptomic space. HisToGene (Pang et al. 2021) adopts a Vision Transformer to capture contextual relationships among image patches. His2ST (Zeng et al. 2022) further incorporates graph neural networks to model spatial dependencies among spots. THItoGene (Jia et al. 2023) combines dynamic convolution and capsule networks to learn morphology-aware molecular signatures, while HGGEP (Li et al. 2024) uses hypergraph neural networks to aggregate multi-stage image features. More recently, mclSTExp (Min et al. 2024) and Reg2ST (Wang et al. 2025) introduced multimodal contrastive learning and more flexible spot relationship modeling, respectively. In related directions, STMCL (Shi et al. 2025b), HISTEX (Xue et al. 2025), HisHRST (Shi et al. 2025a), and SpaICL (Zhao and Min 2025) extend spatial transcriptomics modeling to multi-slice prediction, super-resolution reconstruction, high-density expression generation, and spatial domain identification. Recent studies have also begun to consider robustness in spatial transcriptomics modeling; for example, SpaMask (Min et al. 2025) adopts masking-based graph representation learning to improve stability under sparse data. Despite these advances, existing methods still make limited use of biological priors such as cell-type composition, often rely on restricted visual representations, and insufficiently model complex structural and functional relationships among spots.

However, the aforementioned approaches exhibit several limitations. Firstly, these methods predominantly rely on histology images for prediction while neglecting the critical role of cell type composition. Secondly, they typically employ generic and simplistic feature extraction techniques, which fail to adequately capture the rich and nuanced biological information embedded within the histology images. Thirdly, these methods either underutilize spatial information or merely consider simple spatial proximity between spots, overlooking deeper and more complex spatial relationships. This necessitates the adoption of more sophisticated graph-based structural methods.

To address these limitations, we introduce GR2ST, a deep learning framework designed to capture latent associations between image and gene expression data through contrastive learning and achieves adaptive gene expression generation via a Cell-Type Guided Multi-Branch regression head integrated with cell-type information.

**Figure 1:**
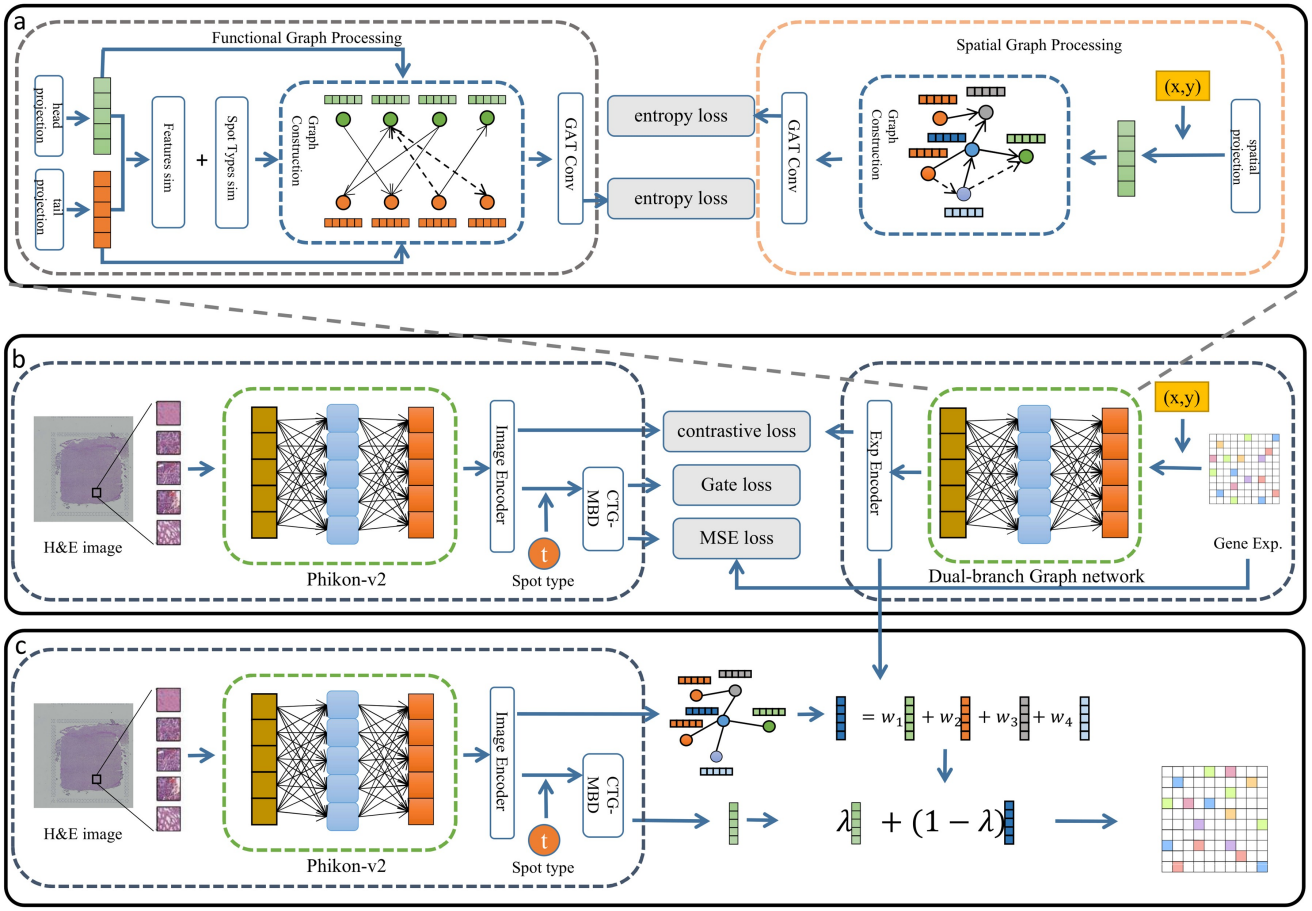
The architecture of GR2ST. (a) Dual-branch Graph Network: for the input gene expression, we use two strategies to construct different graph structures to integrate gene expression. (b) The training process of GR2ST: separately obtain the embedded representations of image features and enhanced gene expression, then use contrastive loss to optimize the alignment of positive sample pairs. Simultaneously, a Cell-Type Guided Multi-Branch Decoder (CTG-MBD) is trained to adaptively generate gene expression, supervised by a cell-type-aware routing mechanism. (c) The testing process of GR2ST: Image patches are mapped into the shape embedding space to retrieve expressions from the k-nearest neighboring spots. The final gene expression is derived by integrating the weighted aggregation of retrieved expressions with the predictions from the CTG-MBD.

Figure 1 depicts the overall architecture of GR2ST, which comprises three integral components: a feature extraction module, a contrastive learning module, and a prediction module. Within the feature extraction module, we employ two distinct strategies to process image and gene expression. For histology images, we utilize a large pre-trained model to extract high-level image features. For gene expression, we designed a dual-branch graph processing component to integrate the gene expression embeddings of each spot, incorporating both functional similarity and spatial proximity. In the contrastive learning module, the extracted image features and gene expression are projected into a shared space. The cosine similarity between these two types of embeddings is computed to minimize their distance within this common space. In the prediction module, inference is achieved through the synergy of a generative path and a retrieval path. On one hand, the model employs a Cell-Type Guided Multi-Branch system to adaptively generate gene expression predictions directly from image embeddings. On the other hand, the target image is projected into the aligned space to retrieve similar spots from the training data for weighted aggregation. Finally, accurate spatial transcriptomics predictions are obtained by integrating the outputs of these two pathways.

## 2 Methods and materials

### 2.1 Datasets and preprocessing

The datasets adopted in this study include the human HER2-positive breast cancer cohort (HER2+) (Andersson et al. 2021), the human cutaneous squamous cell carcinoma (cSCC) collection (Ji et al. 2020), and an additional breast cancer dataset (Alex) (Janesick et al. 2023). The HER2+ dataset contains 36 tissue samples, the cSCC dataset is composed of 12 samples, and the Alex dataset includes 6 breast cancer tissue samples obtained from Swarbrick’s laboratory. All datasets are accompanied by corresponding histology images, spatial coordinates, and matrices of gene expression. For the HER2+ dataset, we retained 32 sections where the number of spots exceeded 180.

A standardized preprocessing workflow was employed across all datasets. For gene expression preprocessing, the 1000 most highly variable genes (HVGs) were identified within each individual tissue section. For each dataset, the final gene set was defined as the intersection of the section-wise HVG sets. In addition, genes detected in fewer than 1000 spots across all sections of the same dataset were excluded to reduce low-expression noise. Counts were normalized by library size, scaled to counts per million (CPM), and log-transformed as log(CPM + 1). After preprocessing, the HER2+, cSCC, and Alex datasets contained 785, 171, and 50 genes, and 9612, 6630, and 25 914 spots, respectively.

For the histology images, each section was partitioned into small patches of 224 × 224 pixels, aligned with the spatial location of every spot. Each patch underwent Reinhard color normalization (Reinhard et al. 2001) to minimize color variations arising from different staining batches, thereby ensuring a consistent staining appearance across all images. Subsequently, for each patch, nuclear type data was extracted using the HoVerNet model (Graham et al. 2019), which had been previously pre-trained on the PanNuke benchmark (Gamper et al. 2019). HoVerNet detects individual nuclei within each patch and classifies them into one of six categories: inflammatory, dead, connective, non-neoplastic epithelial, neoplastic, and unlabeled. A majority voting strategy was then employed to assign a dominant nuclear type to each spot, based on the most frequently predicted nuclear category.

### 2.2 Overview of GR2ST

Following the conceptual overview provided in the Introduction, this section focuses on the technical formulation of GR2ST. Given a spatial transcriptomics sample with n spots, the framework takes as input histology image patches, gene expression profiles, spatial coordinates, and spot-level cell-type annotations. GR2ST consists of three components: (i) a feature extraction module that encodes image and transcriptomic information, (ii) a contrastive learning module that aligns the two modalities in a shared latent space, and (iii) a prediction module that combines adaptive generation and reference-based retrieval for gene expression prediction. The detailed implementation of these components is presented in Sections 2.3–2.5.

### 2.3 Feature extraction module

During the data preprocessing, the image was partitioned into 224 × 224 pixel tiles, with the division guided by the known spatial locations of every spot within the tissue section. As illustrated in Fig. 1b, for each patch, we deployed Phikon-v2 (Filiot et al. 2024), a large-scale pre-trained foundation model to extract image features. Phikon-v2 is a self-supervised learning model specifically designed for histology image analysis. We posit that Phikon-v2 can yield superior performance in this study compared to alternative models. The extracted image features were subsequently projected into a latent space via an image encoder, implemented as a Multilayer Perceptron (MLP). This entire process is formalized in Equation (1).


(1) $$z_{i}^{\mathrm{img}}=\mathrm{MLP}(\mathrm{Phikonv}2({\mathrm{patch}}_{i}))$$

where $\mathrm{patc}h_{i}\in R^{C*H*W}$ represents the image patch of spot i, and$z_{i}^{\mathrm{img}}$ represents the resulting image feature embedding of spot i.

For the gene expression, we integrated it with cell-type embeddings to obtain an augmented gene expression feature, as formalized in Equation (2).


(2) $$e_{i}^{\;\text{exp}\;}\;=\text{exp}+e_{i}^{\mathrm{type}}$$

where $e_{i}^{\mathrm{type}}$ denotes type embedding of spot i, respectively, and $e_{i}^{\;\text{exp}\;}$ represents the augmented gene expression feature.

Graph-based deep learning methods have demonstrated remarkable performance in encoding representations and capturing intrinsic interactions, garnering significant attention in digital pathology (Ahmedt-Aristizabal et al. 2022). Accordingly, we employ a graph-based deep learning approach to update the features of each spot, as illustrated in Fig. 1a. Specifically, we utilize two distinct graph construction strategies—a functional graph pathway and a spatial graph pathway—to account for functional similarity and spatial proximity between spots, respectively. The updated gene expressions from these two pathways are then fused to obtain the final gene representation.

In the functional graph pathway, we adopted a strategy similar to WIKG (Li et al. 2024). Specifically, the augmented gene expression feature of each spot is projected by two independent linear layers to obtain the head-node embedding and the tail-node embedding, respectively, as formalized in Equation (3).


(3) $$h_{i}^{\mathrm{head}}=W_{\mathrm{head}}e_{i}^{\;\text{exp}\;},h_{i}^{\mathrm{tail}}=W_{\mathrm{tail}}e_{i}^{\;\text{exp}\;}$$

where $e_{i}^{\;\text{exp}\;}$ denotes the augmented gene expression feature of spot i, $h_{i}^{\mathrm{head}}$ and $h_{i}^{\mathrm{tail}}$ denote the head-node and tail-node embeddings of spot i, respectively, and $W_{\mathrm{head}}$ and $W_{\mathrm{tail}}$ are learnable projection matrices. Here, i indexes a spot.

For each head-node embedding, we compute its similarity with every tail-node embedding in the same tissue section to quantify functional relationships between spots, as shown in Equation (4).


(4) $$S_{ij}^{\mathrm{feat}}=\frac{〈h_{i}^{\mathrm{head}},h_{j}^{\mathrm{tail}}〉}{\tau }$$

where $S_{ij}^{\mathrm{feat}}$ denotes the feature-based similarity score between spots i and j,$h_{i}^{\mathrm{head}}$ is the head-node embedding of spot i, $h_{j}^{\mathrm{tail}}$ is the tail-node embedding of spot j, ⟨·,·⟩ denotes the inner product operation, and τ is a temperature hyperparameter used to scale the similarity scores.

Simultaneously, we incorporate the influence of spot type on functional interactions by computing type similarity between each pair of nodes, as given in Equation (5).


(5) Sijtype=δ(ci,cj)={1 if ci=cj0 otherwise

where $c_{i}$ represents the cell type of spot i.

Finally, we combine both factors to derive the integrated similarity matrix, which serves as the edge weights between nodes. Nodes with an integrated score exceeding a predefined threshold are selected as neighbors, as formalized in Equations (6) and (7).


(6) $$S_{ij}^{\mathrm{fun}}=S_{ij}^{\mathrm{feat}}+\alpha S_{ij}^{\mathrm{type}}$$

(7) $$N(i)=\{t\in V|S_{it}^{\mathrm{fun}}>=\tau \}$$

where α is a tunable weighting coefficient, |V|=n, |N(i)|=k, n represents the spot count, and N(i) refers to the set of neighboring nodes of node i. τ represents the integrated score.

Since independent projection layers are used for the head and tail nodes, the similarity calculation yields an asymmetric result, thereby facilitating the construction of a directed graph.

In the spatial graph pathway, we use spot coordinates to establish structural connectivity and capture spatial proximity between nodes. Specifically, we calculate the Euclidean distance between each pair of spots according to their spatial coordinates. Instead of using a fixed k-nearest-neighbor strategy, we adopt a radius-constrained rule, where only spots within a spatial distance smaller than R are connected, as formalized in Equations (8) and (9).


(8) $$d_{ij}={\left\|p_{i}-p_{j}\right\|}_{2}$$

(9) Aitspa={1 if dit<R  0 otherwise 

where $A_{it}^{\mathrm{spa}}$ is the adjacency indicator in the spatial graph between spots i and t, $d_{it}$ denotes the Euclidean distance between the two spots with $p_{i}$ and $p_{j}$ representing their respective spatial coordinate vectors, and R is the radius threshold that determines whether an edge is established.

Through the above procedures, we obtain both a functional graph and a spatial graph. Each graph is then processed through a Graph Attention Network (GAT) (Velickovic et al. 2018) to adaptively aggregate neighborhood information and update node features. The update process for node i in the functional graph is formalized in Equations (10)–(12):


(10) $$e_{ij}\;=\;\mathrm{LeakyReLu}(a_{f}^{T}[W_{f}h_{i}||W_{f}h_{j}])$$

(11) $$\alpha_{ij}\;=\;\frac{exp(e_{ij})}{\sum_{k\in N_{f}(i)}\;\text{exp}(e_{ik})}$$

(12) $$h_{i}^{\mathrm{fun}}\;=\;\sigma (\sum_{j\in N_{f}(i)}\alpha_{ij}W_{f}h_{j})$$

where $W_{f}$ is a learnable weight matrix for linear projection of input features; a is the weight vector of the functional graph attention mechanism; N(i) signifies the local neighborhood of node i; σ is a nonlinear activation function; $\alpha_{ij}$ denotes the computed normalized attention weight; and $h_{i}^{\mathrm{fun}}$ is the updated feature representation of node i.

To enable the model to accurately pinpoint the most biologically relevant key nodes among a vast neighborhood and to effectively mitigate the over-smoothing issue common in deep graph neural networks, we introduce an attention entropy minimization loss. This loss function imposes a sparsity constraint on the attention weight distribution, compelling the model to generate a “sharper” and more concentrated attention profile, as formalized in Equation (13):


(13) $$L_{ent}=-\frac{1}{\left|E\right|}\sum_{(i,j)\in E}\sum_{h=1}^{H}\alpha_{ij}^{\left(h\right)}\log(\alpha_{ij}^{\left(h\right)}+\epsilon )$$

where |E| represents the total number of edges in the graph, H denotes the number of attention heads, and ϵ is a minimal value to ensure numerical stability. By penalizing high-entropy weight distributions, this mechanism forces the model to prioritize the most significant spatial and functional interactions, thereby facilitating the extraction of highly discriminative heterogeneous features

Finally, the outputs from the functional graph pathway and the spatial graph pathway are combined and processed through a fusion module, producing the resulting gene expression embedding, as shown in Equations (14) and (15):


(14) $$h_{i}^{\mathrm{fused}}=h_{i}^{\mathrm{fun}}+h_{i}^{\mathrm{spa}}$$

(15) $$z_{i}^{\;\text{exp}\;}=W_{o}R\mathrm{eLU}(W_{p}h_{i}^{\mathrm{fused}})$$

where $h_{i}^{\mathrm{fused}}$ denotes the fused feature of spot i, $h_{i}^{\mathrm{fun}}$ denotes its functional feature, and $h_{i}^{\mathrm{spa}}$ denotes its spatial feature. $z_{i}^{\exp}$ denotes the predicted gene expression vector of spot i, $W_{p}$ and $W_{o}$are learnable projection matrices, andReLU(⋅) is the rectified linear unit activation function.

### 2.4 Contrastive learning module

As illustrated in Fig. 1b, we employ a contrastive learning framework to align histology images with gene expression by pulling matched image–transcript pairs closer and pushing unmatched pairs apart in the latent space. Specifically, the feature extraction module projects visual and transcriptomic features into a shared embedding space. For each spot, the visual embedding and its corresponding transcriptomic embedding form a positive pair, yielding n positive pairs and $n^{2}-n$ negative pairs in total. The objective is to maximize the cosine similarity of positive pairs while minimizing that of negative pairs. Following CLIP (Radford et al. 2021), we formulate the loss as shown in Equations (16)–(18):


(16) $$L_{\mathrm{con}}\;=\;\frac{1}{2}(L_{\mathrm{img}}\;+L_{\;\text{exp}\;})$$

(17) $$L_{\mathrm{img}}=-\frac{1}{N}(\sum_{i=1}^{N}\log(\frac{\;\text{exp}(\mathrm{sim}(z_{i}^{\mathrm{img}},z_{i}^{\;\text{exp}\;})/\tau )}{\sum_{j=1}^{N}\;\text{exp}(\mathrm{sim}(z_{i}^{\mathrm{img}},z_{j}^{\;\text{exp}\;})/\tau )}))$$

(18) $$L_{\;\text{exp}\;}=-\frac{1}{N}(\sum_{i=1}^{N}\log(\frac{\;\text{exp}(\mathrm{sim}(z_{i}^{\;\text{exp}\;},z_{i}^{\mathrm{img}})/\tau )}{\sum_{j=1}^{N}\;\text{exp}(\mathrm{sim}(z_{i}^{\;\text{exp}\;},z_{j}^{\mathrm{img}})/\tau )}))$$

where $L_{\mathrm{img}}$ corresponds to the objective of retrieving the correct gene expression feature using the image feature as a query, and $L_{\;\text{exp}\;}$ corresponds to the symmetric objective of retrieving the correct image feature using the gene expression feature as a query. sim() denotes the cosine similarity metric, while τ serves as the temperature hyperparameter.

### 2.5 Prediction module

As shown in Fig. 1c, to map histology images into the gene expression space, we adopt a dual-path collaborative prediction scheme that integrates adaptive generation with reference-based retrieval.

In the retrieval path, a test image patch is processed in the same way as in training. Specifically, the patch is encoded by Phikon-v2 and then projected into the shared embedding space:


(19) $$z_{\mathrm{test}}^{\mathrm{img}}=\mathrm{MLP}(\mathrm{Phikonv}2({\mathrm{patch}}_{\mathrm{test}}))$$

where ${\mathrm{patch}}_{\mathrm{test}}$ denotes the input histology patch of the test spot, Phikonv2(⋅) is the pre-trained pathology foundation model used for image feature extraction, MLP(⋅) is the image projection encoder, and $z_{\mathrm{test}}^{\mathrm{img}}$ denotes the image embedding of the test spot in the shared latent space.

Next, we compute the semantic similarity between the test image embedding and each reference gene-expression embedding in the training set. Based on these similarity scores, the top-k most similar reference spots are selected to form the retrieval set:


(20) $$N(z_{\mathrm{test}}^{\mathrm{img}})=\{t\in V|\sigma (z_{t}^{\;\text{exp}\;})\in \mathrm{TopK}{\left\{\sigma (z_{i}^{\;\text{exp}\;})\right\}}_{i=1}^{n}\}$$

(21) $$\sigma (z)=\mathrm{sim}(z_{\mathrm{test}}^{\mathrm{img}},\;z)$$

where V denotes the set of reference spots in the training database, $z_{t}^{\exp}$ is the gene-expression embedding of reference spot t, sim(⋅,⋅) denotes the cosine similarity function,$\sigma (z_{t}^{\exp})$ is the similarity score between the test image embedding and the embedding of reference spot t, and $N(z_{\mathrm{test}}^{\mathrm{img}})$denotes the set of the top-k retrieved reference spots.

For each reference spot $t\in N(z_{\mathrm{test}}^{\mathrm{img}})$, we further compute its Manhattan distance to the query image embedding:


(22) $$d_{t}={\left|z_{\mathrm{test}}^{\mathrm{img}}-z_{t}^{\;\text{exp}\;}\right|}_{1}$$

where $d_{t}$ denotes the Manhattan distance between the test image embedding and the gene-expression embedding of reference spot t, and $\|\cdot {\|}_{1}$ denotes the$L_{1}$ norm.

The weight of each retrieved reference spot is then defined as the reciprocal of the squared distance:


(23) $$w_{t}=\frac{1}{d_{t}^{2}}$$

where $w_{t}$ denotes the unnormalized retrieval weight assigned to reference spot t.

These weights are normalized to ensure that they sum to 1:


(24) $${\hat{w}}_{t}=\frac{w_{t}}{\sum_{t\in N(z_{\mathrm{test}}^{\mathrm{img}})}w_{t}}$$

where ${\hat{w}}_{t}$ denotes the normalized retrieval weight of reference spot t.

Finally, the retrieval-based transcriptomic prediction for the test image is obtained by weighted aggregation of the reference expression vectors:


(25) $${\hat{y}}_{\mathrm{ret}}=\sum_{t\in N(z_{\mathrm{test}}^{\mathrm{img}})}{\hat{w}}_{t}y_{t}$$

where $y_{t}$ represents the true gene expression vector of reference spot t.

In the generative path, the image embedding is fed into the Cell-Type Guided Multi-Branch Decoder (CTG-MBD). Instead of using a single decoder, CTG-MBD consists of k parallel phenotype-specific MLP branches, each designed to model the transcriptomic pattern of a distinct cellular phenotype.

A cell-type-aware routing mechanism is introduced to coordinate these branches. It maps the image embedding to a soft probability vector that determines the contribution of each branch. Unlike implicit attention mechanisms, the router is explicitly supervised by cell-type annotations through a cross-entropy loss, guiding it to identify the spot’s biological identity and activate the appropriate expert branches.

Formally, the routing weights w and the generative prediction ${\hat{y}}_{\mathrm{gen}}$ are calculated as follows:


(26) $$w=\mathrm{Softmax}({\mathrm{MLP}}_{\mathrm{router}}(z_{\mathrm{test}}^{\mathrm{img}}))$$

(27) $${\hat{y}}_{\mathrm{gen}}=\sum_{k=1}^{K}w_{k}\cdot B_{k}(z_{\mathrm{test}}^{\mathrm{img}})$$

where $B_{k}$ denotes the output of the k-th regression branch. Through this cell-type-aware modulation, the CTG-MBD achieves adaptive gene expression generation that dynamically aligns with the local cellular heterogeneity.

The final gene expression prediction $\hat{y}$ is derived by integrating the outputs of these two pathways:


(28) $$\hat{y}=\lambda \cdot {\hat{y}}_{\mathrm{ret}}+(1-\lambda )\cdot {\hat{y}}_{\mathrm{gen}}$$

where λ is a balancing coefficient (set to 0.4 in this study) that controls the contribution of retrieval and generation.

### 2.6 Optimization objective

To train GR2ST effectively, we design a multi-task objective function that jointly optimizes the multimodal alignment, the generative accuracy, and the biological consistency of the routing mechanism. First, to ensure the generative path accurately reconstructs gene expression values, we employ the Mean Squared Error (MSE) loss between the output of the CTG-MBD and the ground truth expression:


(29) $$L_{\mathrm{mse}}=\frac{1}{N}\sum_{i=1}^{N}\|y_{i}-{\hat{y}}_{\mathrm{gen},i}{\|}_{2}^{2}$$

where $y_{i}$ and ${\hat{y}}_{\mathrm{gen},i}$ denote the ground truth vector and the generative prediction for the *i*-th spot, respectively.

Second, as mentioned above, to enforce the semantic meaning of the routing weights, we impose an explicit supervision on the routing network using the cell-type labels derived from HoVerNet. This is formulated as a Cross-Entropy loss:


(30) $$L_{\mathrm{gate}}=-\frac{1}{N}\sum_{i=1}^{N}\sum_{c=1}^{C}y_{\mathrm{type},i}^{\left(c\right)}\log(w_{i}^{\left(c\right)})$$

where C signifies the total count of cell categories (set to 6), $y_{\mathrm{type}}$ constitutes the one-hot encoded cell type, and w is the predicted probability distribution from the routing mechanism.

Finally, the overall training objective is a weighted combination of the contrastive loss (defined in Section 2.4), the regression loss, the gate loss, and the attention entropy loss:


(31) $$L_{\mathrm{total}}=L_{\mathrm{con}}+\alpha_{\mathrm{mse}}L_{\mathrm{mse}}+\alpha_{\mathrm{gate}}L_{\mathrm{gate}}+\alpha_{\mathrm{ent}}L_{\mathrm{ent}}$$

where $\alpha_{\mathrm{mse}}$, $\alpha_{\mathrm{gate}}$, and $\alpha_{\mathrm{ent}}$ are hyperparameters controlling the trade-off between different tasks.

## 3 Experiments and results

### 3.1 Evaluated metrics and criteria

To assess the linear relationship between the estimated and actual gene expression profiles, we utilized the Pearson Correlation Coefficient (PCC). PCC values range between -1 and 1, where an absolute value closer to 1 indicates a stronger linear association, and a value nearer to 0 suggests a weaker one. The PCC is computed as follows:


(32) $$\mathrm{PCC}=\frac{\mathrm{Cov}(X_{\mathrm{true}},X_{\mathrm{pred}})}{\sigma (X_{\mathrm{true}})\sigma (X_{\mathrm{pred}})}$$

where Cov() signifies covariance, $X_{\mathrm{true}}$ and $X_{\mathrm{pred}}$ represent the true and predicted gene expression, σ represents the standard deviation.

### 3.2 Experiment

We assessed our model’s predictive capability by comparing it with seven existing leading methods for spatial transcriptomics prediction: STMCL, Reg2ST, mclSTExp, HGGEP, THItoGene, Hist2ST, and HisToGene. The evaluation employed a leave-one-out cross-validation scheme; in every round, a single tissue section was reserved for testing, while all others formed the training set. This procedure was performed on the cSCC collection (comprising 12 sections), the HER2+ cohort (comprising 32 sections) and the Alex dataset (comprising 6 sections).

The results of all models on three datasets are presented in Table 1. Among the top three methods on the HER2+ dataset, GR2ST achieved an average PCC that was 3.33% higher than mclSTExp and 2.52% higher than STMCL, and reducing the prediction error with the lowest MAE of 0.5733. On the cSCC dataset, GR2ST outperformed mclSTExp by 4.14% and STMCL by 2.46% within the top three approaches. Furthermore, the comparable MAE and MSE values on the cSCC dataset indicate that GR2ST achieves high-fidelity spatial mapping without compromising numerical stability. On the Alex dataset, GR2ST achieved an average PCC that was 35.5% higher than Reg2ST and 24.86% higher than STMCL, and reducing the prediction error with the lowest MAE of 0.3897 and lowest MSE of 0.2565.

**Table 1:** Results of comparative experiments.

Model	HER2+			cSCC			Alex		
	PCC	MAE	MSE	PCC	MAE	MSE	PCC	MAE	MSE
GR2ST	**0.2357**	**0.5733**	0.6427	**0.3288**	0.5390	0.4717	**0.1130**	**0.3897**	**0.2565**
HisToGene	0.0818	0.6422	0.5202	0.0771	0.6234	0.5223	0.0392	0.4201	0.2773
THItoGene	0.1330	0.5958	**0.5012**	0.1796	0.6012	0.5021	0.0638	0.4109	0.2966
Hist2ST	0.1484	0.6087	0.5135	0.1749	0.6107	0.5103	0.0712	0.4211	0.2954
HGGEP	0.1566	0.5824	0.6015	0.1084	0.5399	0.5163	0.0751	0.4287	0.2878
mclSTExp	0.2281	0.5877	0.6020	0.3157	0.5272	0.4403	0.0778	0.4003	0.2601
Reg2ST	0.1741	0.5911	0.5678	0.2024	0.5477	0.5032	0.0834	0.3987	0.2667
STMCL	0.2299	0.5907	0.6002	0.3209	**0.5112**	**0.4332**	0.0905	0.4054	0.2657

Bold values indicate the best performance, and underlined values indicate the second-best performance.

To determine whether the improvements of GR2ST over baseline methods were statistically significant, a Wilcoxon signed-rank test was performed on the PCC values obtained from predicted and ground truth gene expression for every spot within the cSCC, Alex and HER2+ datasets. Specifically, for each tissue section in each dataset, the differences in PCC between GR2ST and each baseline method were computed. This test examined if the median difference was statistically greater than zero. The results are summarized in Table 2. It can be observed that GR2ST demonstrates substantially superior performance over all other methods on the datasets.

**Table 2:** Results of Wilcoxon signed-rank test.

Model	HER2+			cSCC			Alex		
	Median diff	Wilcoxon *P*-value	Significance (α = 0.05)	Median diff	Wilcoxon *P*-value	Significance (α = 0.05)	Median diff	Wilcoxon *P*-value	Significance (α = 0.05)
HisToGene	0.1323	< 0.0001	Yes	0.2543	0.0002	Yes	0.0648	< 0.0001	Yes
THItoGene	0.0984	< 0.0001	Yes	0.1607	0.0002	Yes	0.0432	< 0.0001	Yes
Hist2ST	0.0896	< 0.0001	Yes	0.1567	0.0002	Yes	0.0367	< 0.0001	Yes
HGGEP	0.0849	< 0.0001	Yes	0.2088	0.0002	Yes	0.0333	< 0.0001	Yes
mclSTExp	0.0066	< 0.0001	Yes	0.0114	0.0081	Yes	0.0394	0.0156	Yes
Reg2ST	0.0597	< 0.0001	Yes	0.1403	0.0002	Yes	0.0259	< 0.0001	Yes
STMCL	0.0257	< 0.0001	Yes	0.0090	0.0647	No	0.0231	0.0469	Yes

**Figure 2:**
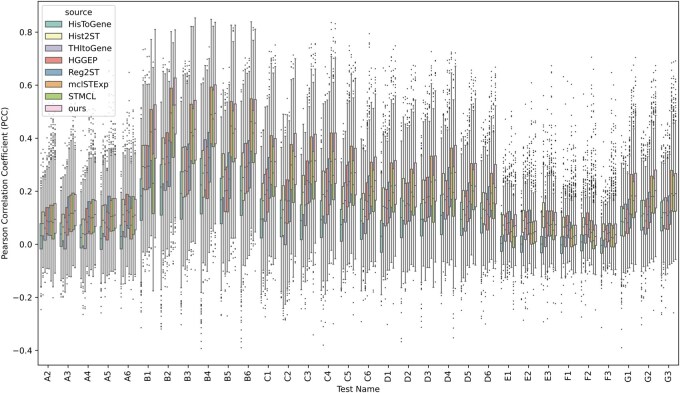
Comparative experimental results on HER2+ dataset. Performance on the HER2+ cohort was assessed by calculating the PCC between ground truth and estimated gene expression for several models, including HisToGene, His2ST, THItoGene, HGGEP, Reg2ST, mclSTExp, STMCL and GR2ST. High-resolution images are accessible at https://github.com/zjl1109294570/GR2ST/blob/main/GR2ST/results/her2st_output.pdf.

Subsequently, we visualized the PCC for each section to examine the results, as shown in Fig. 2 and Figs. S1 and S2 (see Supplementary Note 1).

Additional ablation studies evaluating the effects of the functional graph, spatial graph, cell-type information, and image feature extractor are provided in the Supplementary Materials (Supplementary Note 2, Figs. S3–S5).

We further evaluated the sensitivity of GR2ST to several key hyperparameters on the HER2+ dataset, including α_MSE, projection dimension, spatial radius R, and confidence threshold δ. The detailed results are provided in Supplementary Note 4 and Fig. S7.

The construction of the functional graph requires global pairwise similarity computation among spots and therefore has a theoretical complexity of O(n^2^), where n denotes the number of spots in a tissue section. To assess the practical overhead, we profiled the inference runtime and GPU memory usage on the HER2+ dataset using trained models. Across tissue sections containing 176–712 spots, the average total inference time was approximately 0.008 s per section, while the functional graph construction required about 0.0003–0.0008 s per section. Peak GPU memory usage ranged from 0.61 GB to 4.07 GB depending on slice size. These results indicate that the current implementation is computationally feasible for the moderate-scale sections used in this study. For substantially larger tissue slices, future optimization may include approximate nearest-neighbor search, sparse top-k graph construction, or block-wise similarity computation. All runtime measurements were obtained on a workstation equipped with an NVIDIA GPU RTX A6000 and PyTorch implementation.

We further evaluated robustness under random image masking, Gaussian noise, and graph edge dropout perturbations. As shown in Figs. S8–S10, the model degrades gradually under image-level corruption while remaining highly stable under graph topology perturbation.

### 3.3 Spatial region detection

To rigorously assess the efficacy of various approaches in dissecting spatial domains within whole-slide H&E images, we conducted a comparative study using six tissue sections from the HER2+ dataset, all of which possess ground-truth annotations provided by pathologists. Upon generating the predicted gene expression profiles, we applied dimensionality reduction techniques followed by clustering algorithms to categorize spots into distinct spatial regions. In contrast to competing methods, GR2ST demonstrated a superior capability to reconstruct spatial architectures that align closely with pathological definitions, yielding substantial improvements in segmentation accuracy.

**Figure 3:**
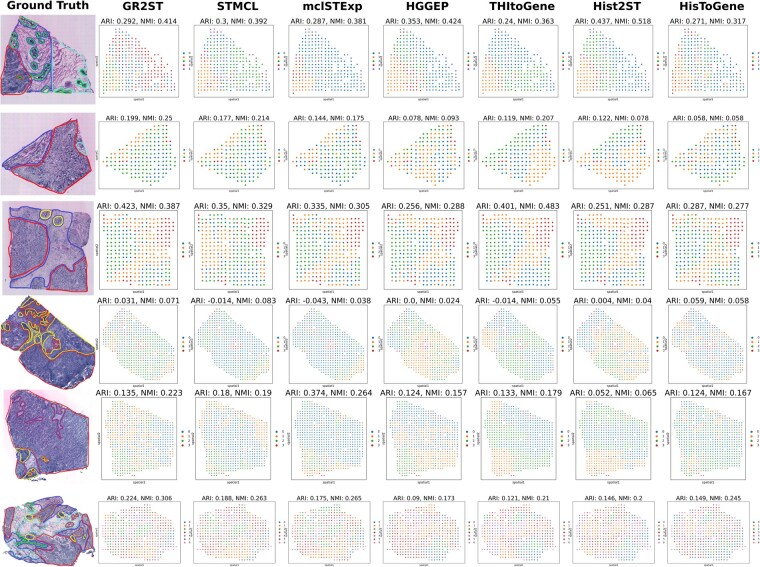
Visualization of spatial domain detection in the HER2+ dataset. The detection is based on the clustering of predicted gene expression profiles, where the performance is quantified using ARI and NMI scores relative to pathologist annotations. The compared methods include STMCL, GR2ST, mclSTExp, HGGEP, THItoGene, Hist2ST, and HisToGene.

As evidenced in Fig. 3, our method consistently secured the leading ARI and NMI scores across the evaluated slices. Quantitatively, GR2ST achieved remarkable performance gains.

## 4 Conclusion

In this study, we focus on the problem of spatial transcriptomics gene prediction to address the current limitations of long sequencing time and high costs in spatial transcriptomics technologies, thereby facilitating further advancement in the field. We introduce GR2ST, a novel multimodal deep learning approach developed by combining graph representation learning with a contrastive learning framework. The spots detected by spatial transcriptomics are treated as graph nodes, with enhanced gene expression profiles serving as node features. Threshold functional and radius spatial graphs are constructed using node features and spot location information, respectively, and feature integration is performed via graph attention mechanisms.

Notably, we innovatively employ a pre-trained large model for image feature extraction and incorporate cell type data to construct a Cell-Type Guided Multi-Branch Decoder (CTG-MBD). We subsequently adopt a contrastive learning strategy to synchronize image features with enhanced gene expression from positive samples, while simultaneously training the decoder for adaptive generation. Finally, the predicted gene expression for test data is obtained by integrating the generative output with the weighted aggregation of the true gene expression from the top-k most similar nodes.

Experiments were conducted on three datasets using seven baseline methods. The results demonstrate that GR2ST outperforms all baseline approaches. Furthermore, spatial domain identification confirms that the model’s predictions effectively reconstruct biologically meaningful tissue structures, validating both the effectiveness and biological relevance of our model.

## Supplementary Material

btag209_Supplementary_Data

## Data Availability

The code for GR2ST is publicly available at https://github.com/zjl1109294570/GR2ST. The datasets analyzed in this study are publicly available from the original publications: the HER2-positive breast cancer dataset (Andersson et al. 2021), the cutaneous squamous cell carcinoma dataset (Ji et al. 2020), and the Alex breast cancer dataset (Janesick et al. 2023).
