# Supplementary material for: GR2ST: spatial transcriptomics prediction based on graph-enhanced multimodal contrastive learning
Source: Bioinformatics. 2026 Apr 26;42(5):btag209. doi: 10.1093/bioinformatics/btag209 (PMC13157223; doi:10.1093/bioinformatics/btag209)
Supplement: btag209_Supplementary_Data [file btag209_supplementary_data.docx]

## Supplementary Materials for

## GR2ST: Spatial Transcriptomics Prediction based on Graph-Enhanced Multimodal Contrastive Learning

**Supplementary Notes**

**Supplementary Note 1: Visualization of the PCC for each section**

We visualized the PCC for each section to examine the results, as shown in Figure 2, Figure S1 and Figure S2. The superiority of our model is evident, as it consistently exceeds the performance of other models in the majority of sections, thereby demonstrating its robust effectiveness.

**Supplementary Note 2: Ablation experiment**

We conducted ablation tests to assess the individual impact of distinct model components . The experiments examine four modifications: removal of the functional graph, removal of the spatial graph, omission of cell type data, and replacement of the large pre-trained model for image feature extraction.

Outcomes are illustrated in Figures S3, Figure S4 and Figure S5. Here, “wo_type” indicates the model without cell type data, “wo_function” denotes removal of the functional graph, “wo_spatial” refers to removal of the spatial graph, and “wo_phikon” indicates that the Phikon-v2 feature extractor was replaced with ResNet (He, et al., 2016).

The results show that removing any component resulted in varying degrees of performance degradation. The most substantial decline occurred when the large pre-trained model was replaced with ResNet for feature extraction, which led to a decrease of 16.8% on the HER2+ dataset, 13.1% on the cSCC dataset and 12.9% on the Alex dataset, demonstrating the effectiveness of Phikon-v2.

**Supplementary Note 3: Visual Assessment of Predicted Expression Profiles**

To provide a deeper evaluation regarding the fidelity of the generated gene expression maps, genes were arranged according to their mean −log10(P-values) computed throughout all sections, with P-values derived from comparisons between predicted and actual expression levels. The seven highest-ranking genes—GNAS, FN1, FASN, SCD, HLA-B, CLDN4, and HLA-DRA—were chosen for further investigation.

The majority of these genes are strongly implicated in breast cancer pathogenesis. FN1 codes for fibronectin—a protein crucial for upholding cellular structure and function, which also functions as a multifunctional regulator within the stromal microenvironment of tumors (Spada, et al., 2021). Upregulation of GNAS in breast carcinoma contexts may indicate a novel research focus for exploring mutated genes in breast cancer (Johannet, et al., 2024). Suppressing FASN leads to programmed cell death in breast cancer cells, indicating a potential treatment approach (Menendez & Lupu, 2017). SCD is pivotal for de novo fatty acid lipogenesis and participates in several essential oncogenic signaling cascades (Sen,U. et al. 2023). CLDN4-based cell adhesion signals can accelerate the metabolism and progression of breast cancer through LXR β (Murakami, et al., 2023). HLA-B expression levels show a strong association with survival and recurrence outcomes in breast cancer (Noblejas-López, et al., 2019), while the expression level of HLA-DRA may offer a new approach for enhancing treatment and improving overall survival in advanced breast cancer (Saraiva, et al., 2021).

In the HER2+ dataset, the expression patterns of these seven genes were visualized. Figure S6 demonstrates that our model produced the highest PCC values for four out of these seven genes, with scores of 0.854, 0.816, 0.77, and 0.81.

**Supplementary Note 4: Sensitivity analysis of key hyperparameters**

To evaluate the robustness of GR2ST with respect to hyperparameter selection, we conducted a sensitivity analysis on four key hyperparameters: the regression-loss weight $\alpha_{MSE}$, the shared projection dimension, the spatial graph radius R, and the confidence threshold δ used in functional graph construction. For each analysis, one hyperparameter was varied while the others were fixed at their default values, and the model performance was assessed using the average Pearson correlation coefficient (Avg PCC).

Specifically, α_MSE was evaluated over {10, 20, 50, 100}, the projection dimension was evaluated over {128, 256, 512}, the spatial radius R was evaluated over {2.0, 2.5, 3.0, 3.5, 4.0}, and the confidence threshold δ was evaluated over {0.4, 0.5, 0.6, 0.7, 0.8}. The results are shown in Supplementary Figure S7.

As shown in Supplementary Figure S7c, the model performance generally improved as α_MSE increased, with the best Avg PCC achieved at α_MSE = 100. This suggests that assigning a relatively larger weight to the regression objective is beneficial for gene expression prediction. For the shared projection dimension (Supplementary Figure S7d), the best performance was obtained at 128, while increasing the dimension to 256 or 512 did not lead to further improvement. This indicates that a compact latent space is sufficient for effective cross-modal representation alignment in GR2ST.

For the spatial graph radius R (Supplementary Figure S7a), the model achieved the best performance at R = 3.5. In contrast, the performance dropped noticeably when R = 3.0, suggesting that an overly restricted spatial neighborhood may limit the aggregation of informative local context. For the confidence threshold δ (Supplementary Figure S7b), the best result was observed at δ = 0.7. Lower thresholds may introduce noisy functional edges, whereas higher thresholds may remove useful associations, leading to suboptimal performance.

Overall, these results indicate that GR2ST is reasonably robust to moderate changes in key hyperparameters. The parameter configurations are summarized in Table S1.

**Supplementary Note 5: Robustness analysis**

To further evaluate the stability of our framework, we conducted additional robustness experiments on the HER2+ dataset under both image-level and graph-level perturbations. All perturbations were applied only during inference, while the trained model parameters and evaluation pipeline remained unchanged. Following the evaluation setting in the main manuscript, we report HVG PCC, MSE, and MAE.

We first evaluated the effect of partial visual information loss by randomly masking image information at increasing ratios. As shown in Figure S8, prediction performance decreases progressively as the masking ratio increases. Specifically, HVG PCC declines while both MSE and MAE increase, indicating that the model is affected by severe image information loss but remains relatively stable under moderate masking perturbations.

We further assessed robustness to feature corruption by adding Gaussian noise with increasing levels to the precomputed image features. As shown in Figure S9, model performance declines consistently as the noise level increases. Compared with random masking, Gaussian noise causes a more pronounced degradation, suggesting that global corruption of image representations is more harmful than localized information removal.

To assess sensitivity to graph topology corruption, we randomly removed edges from the functional graph, the spatial graph, or both during inference. As shown in Figure S10, graph edge dropout causes only marginal performance changes across all settings. The effect is slightly more noticeable when perturbing the spatial graph or both graphs jointly than when perturbing only the functional graph, suggesting that spatial connectivity provides somewhat stronger structural support in this setting. Nevertheless, the overall degradation remains limited even under substantial edge dropout, indicating that the graph module is robust to partial topology corruption.

Overall, these robustness experiments show that the proposed framework degrades gradually under image-level perturbations and remains highly stable under graph-level perturbations. These findings support the reliability of the method in practical scenarios where histology inputs may be partially corrupted and graph construction may be imperfect.

**Supplementary Figures**


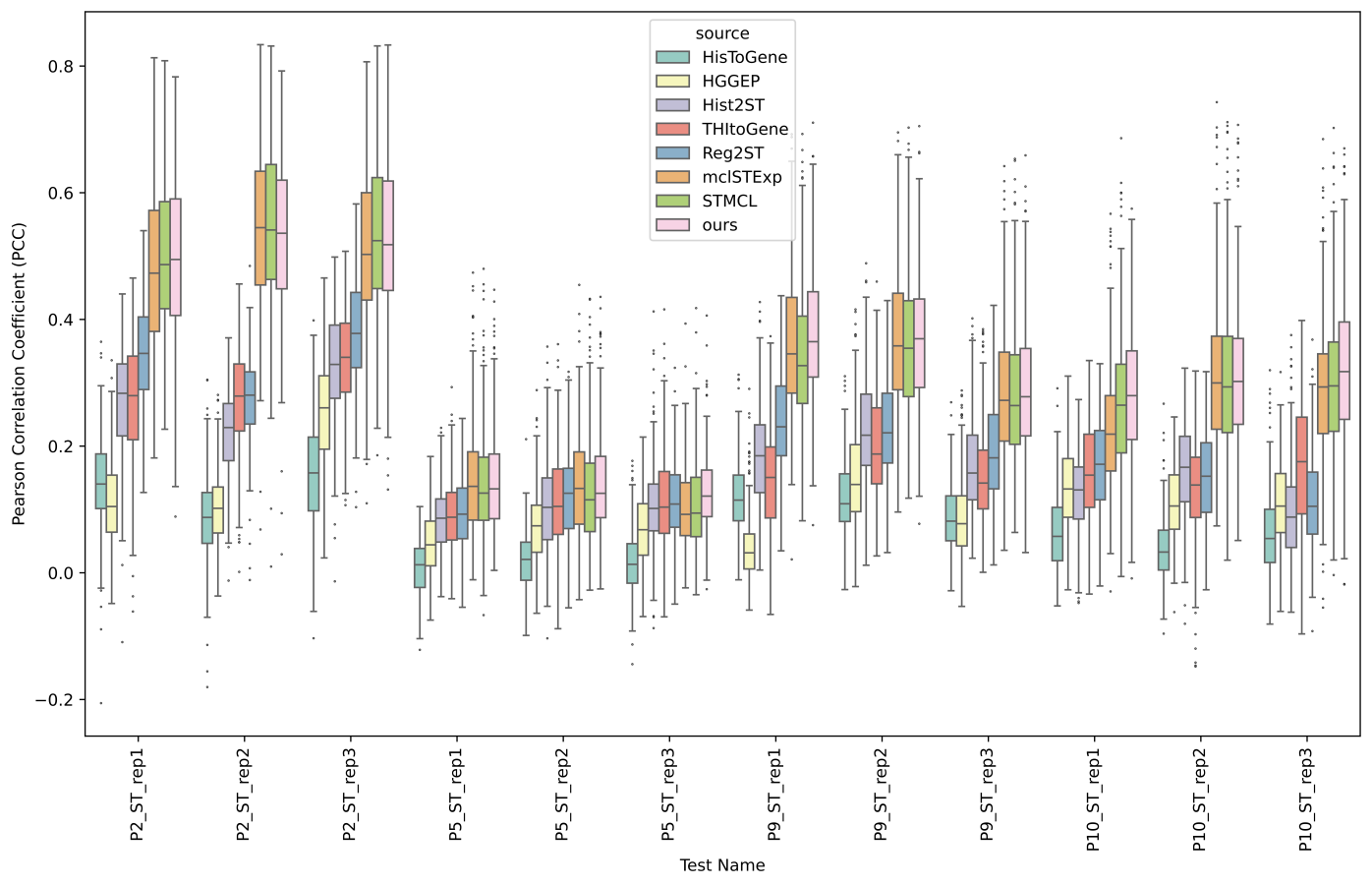


**Fig. S1. Comparative experimental results on cSCC dataset.** Performance on the cSCC dataset was assessed by calculating the PCC between the true and predicted gene expression for several models, including HisToGene, His2ST, THItoGene, HGGEP, Reg2ST, mclSTExp, STMCL and GR2ST. High-resolution images can be obtained at https://github.com/zjl1109294570/GR2ST/blob/main/GR2ST/results/cscc_output.pdf.


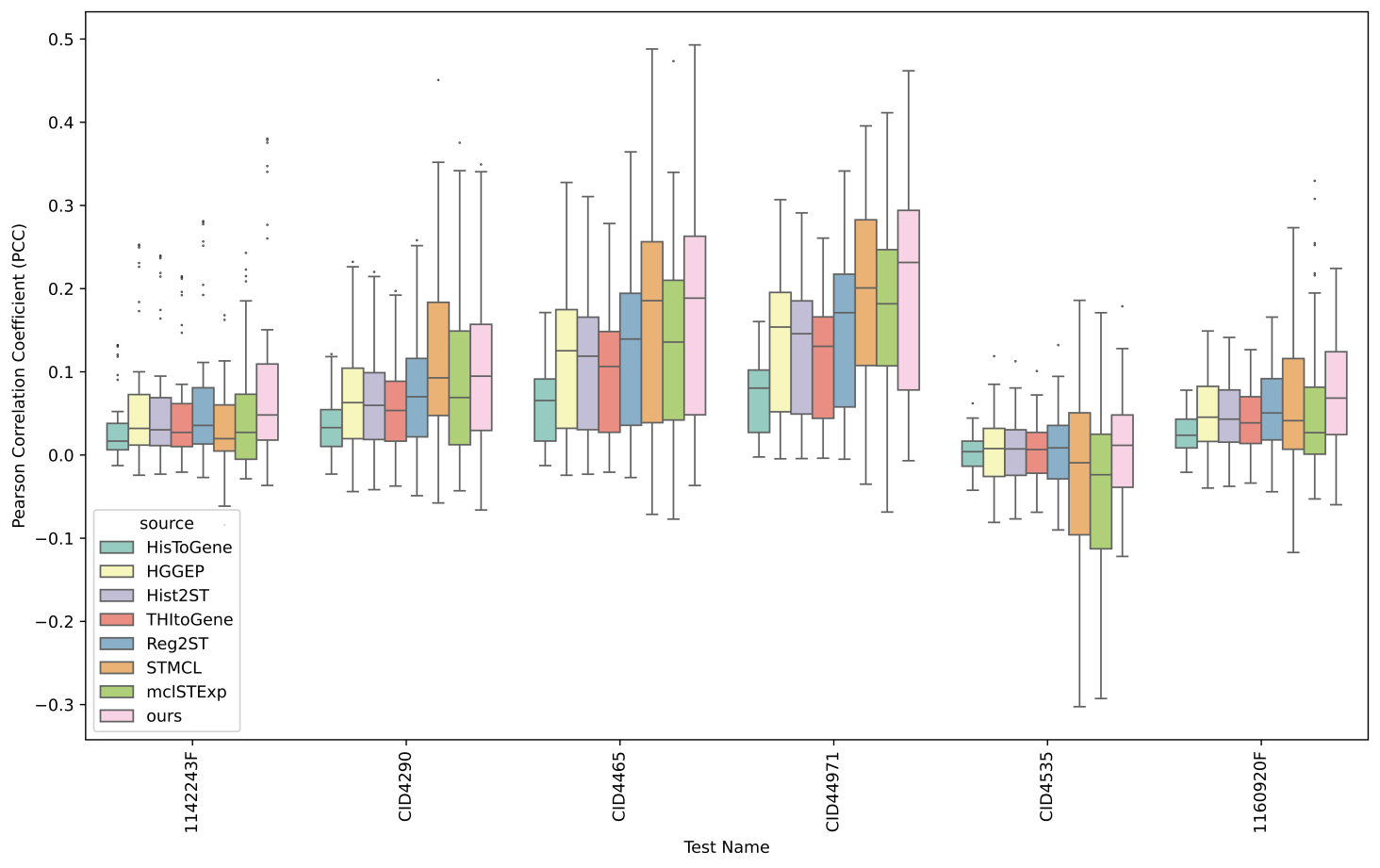


**Fig. S2. Comparative experimental results on Alex dataset.** Performance on the Alex dataset was assessed by calculating the PCC between the true and predicted gene expression for several models, including HisToGene, His2ST, THItoGene, HGGEP, Reg2ST, mclSTExp, STMCL and GR2ST. High-resolution images can be obtained at https://github.com/zjl1109294570/GR2ST/blob/main/GR2ST/results/alex_output.pdf.


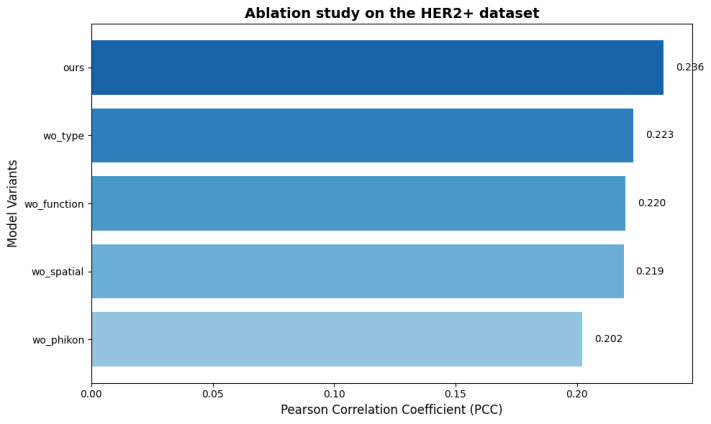


**Fig. S3. Ablation study on HER2+ dataset.** The results are the PCC values of GR2ST after removing specific modules on the HER2+ dataset.


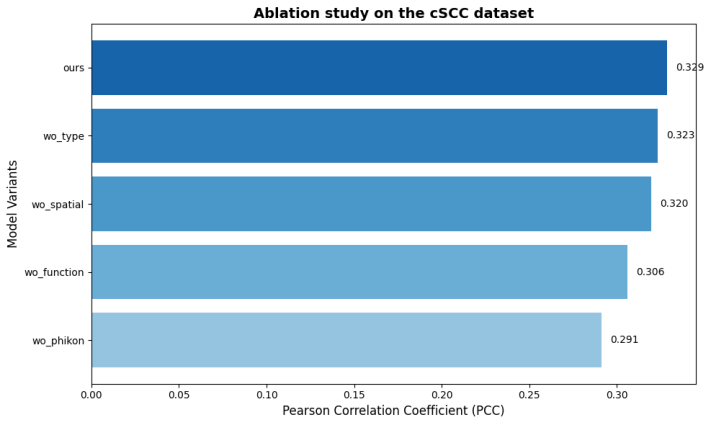


**Fig. S4. Ablation study on cSCC dataset.** The results are the PCC values of GR2ST after removing specific modules on the cSCC dataset.


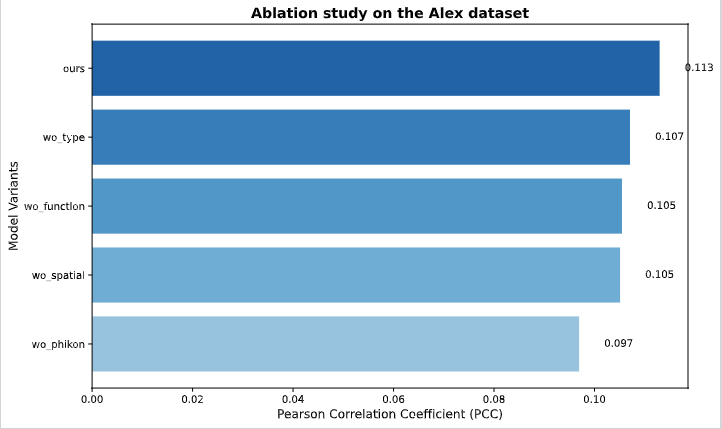


**Fig. S5. Ablation study on Alex dataset.** The results are the PCC values of GR2ST after removing specific modules on the Alex dataset.

**
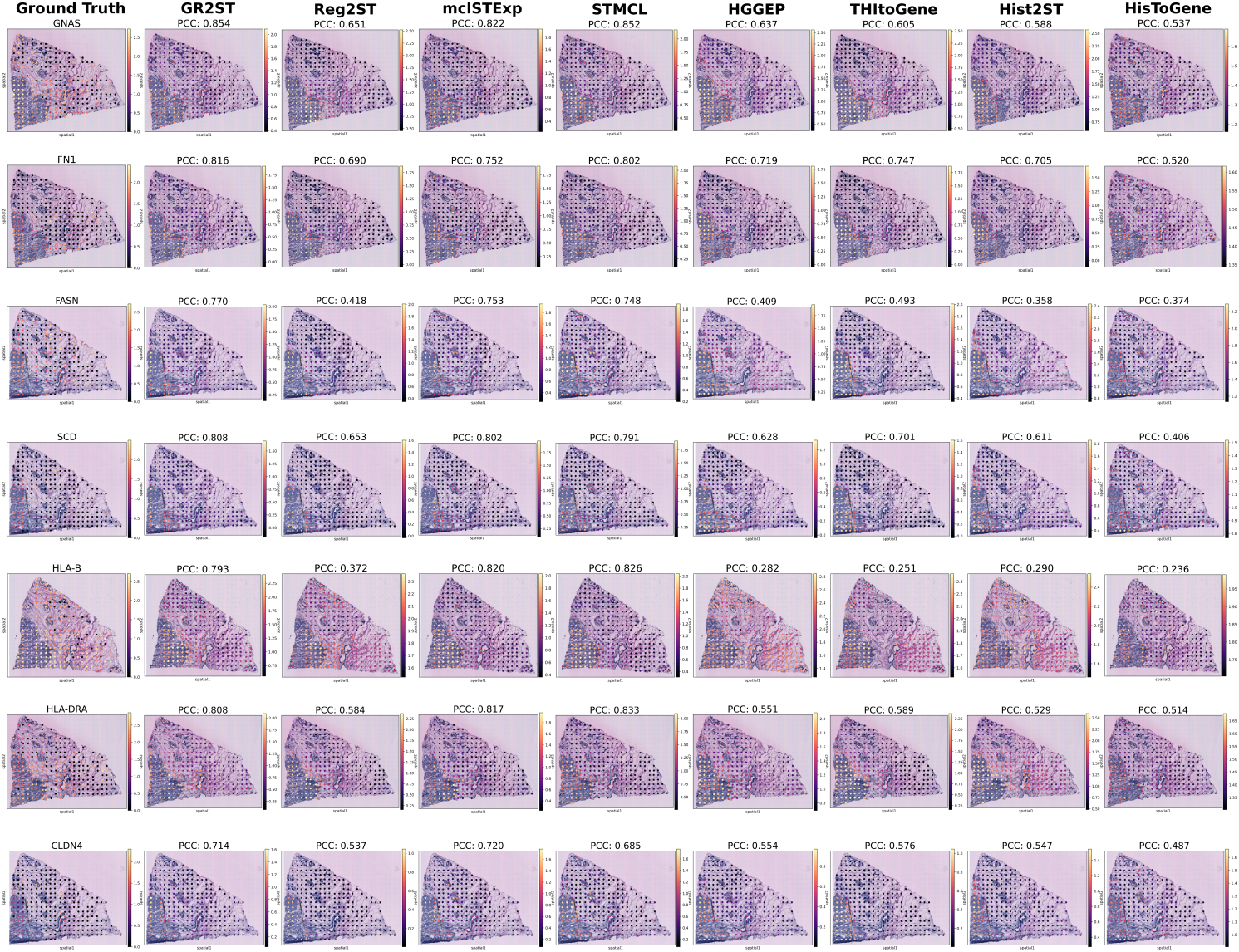
**

**Fig. S6. Visual Assessment of top seven predicted genes in the HER2+ cohort.** The prediction is based on the average -log(P-values) across all tissue sections, where the P-values were computed from the predicted and true gene expression. The top seven genes are GNAS, FN1, FASN, SCD, HLA-B, CLDN4, and HLA-DRA

**
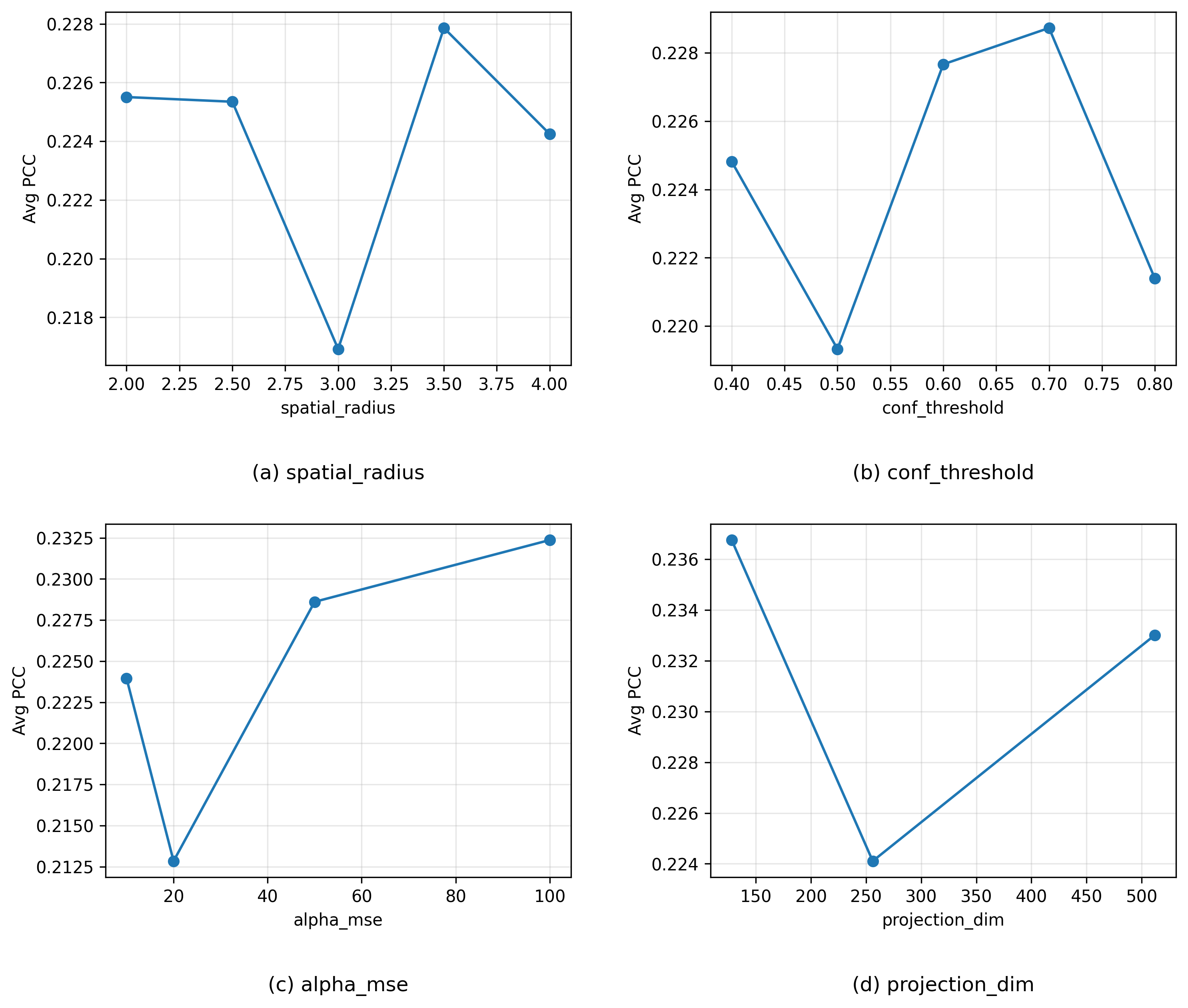
Fig. S7. Sensitivity analysis of key hyperparameters in GR2ST.** (a) Effect of the spatial graph radius R. (b) Effect of the confidence threshold δ used in functional graph construction. (c) Effect of the regression-loss weight α_MSE. (d) Effect of the shared projection dimension. Performance is measured by average Pearson correlation coefficient (Avg PCC).

**
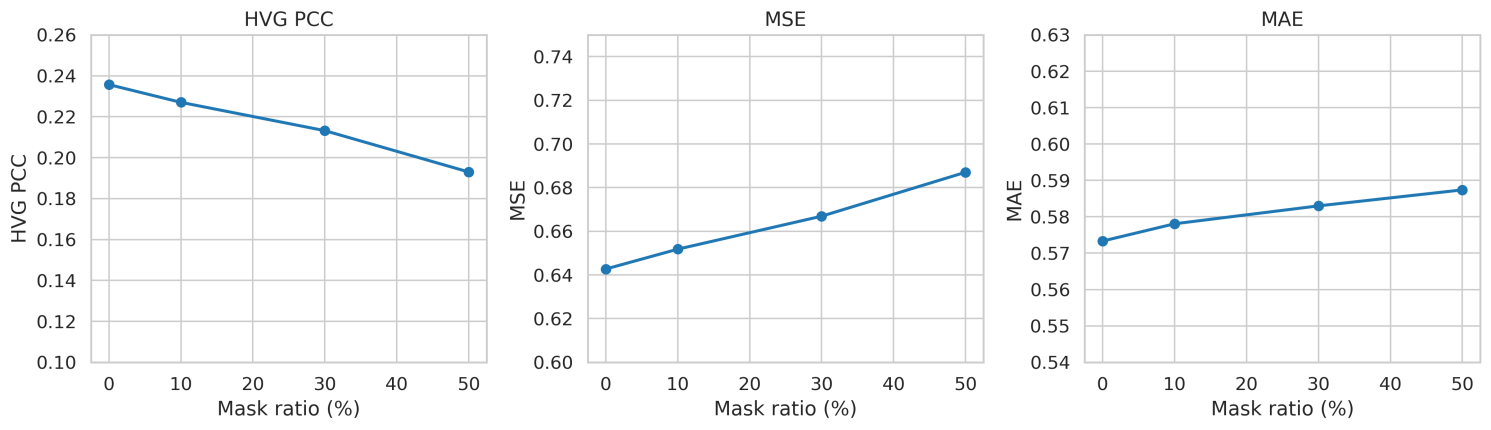
**

**Fig. S8. Robustness to image masking on HER2+ dataset.**

**
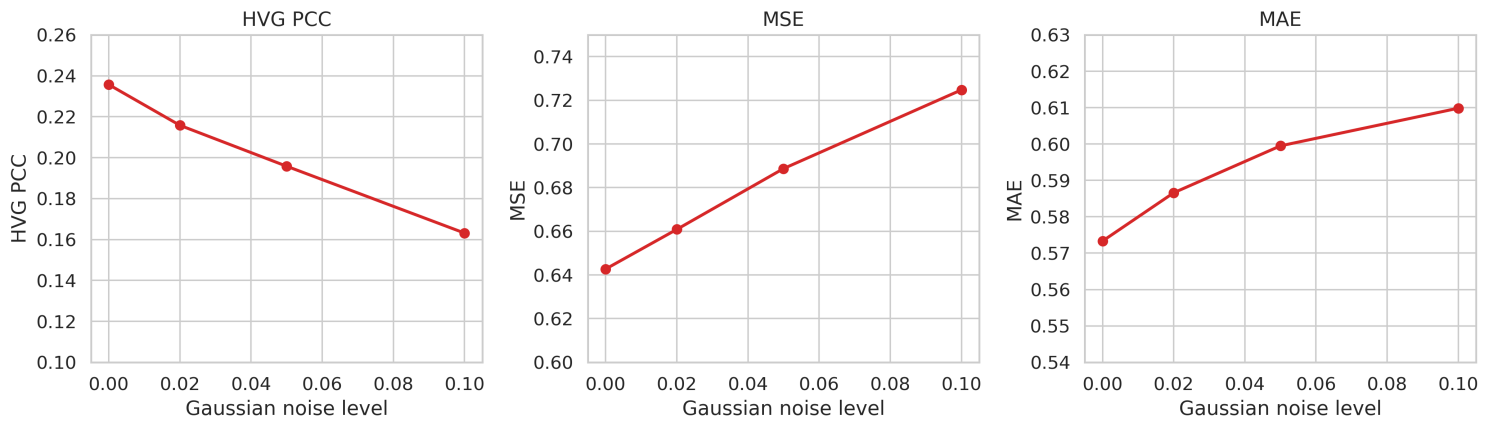
**

**Fig. S9. Robustness to Gaussian noise on HER2+ dataset.**

**
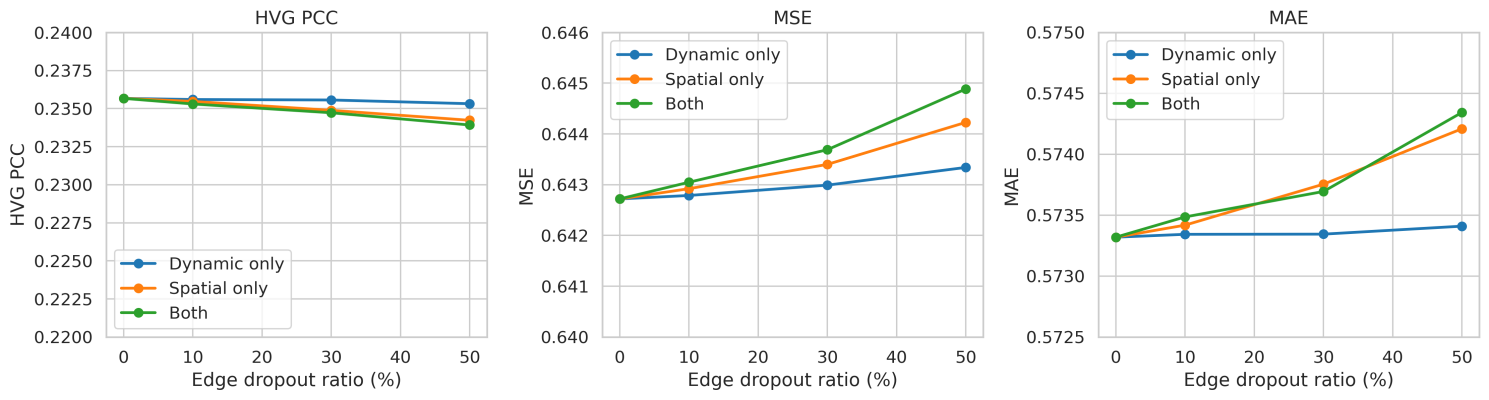
**

**Fig. S10. Robustness to graph edge dropout on HER2+ dataset.**

**Supplementary Tables**

**Table. S1. Hyperparameter Configurations for the Proposed GR2ST Model.**

| **hyperparameters** | **Value** |
| --- | --- |
| projection dimension | 128 |
| the regression-loss weight $\alpha_{MSE}$ | 100 |
| spatial radius R | 3.5 |
| confidence threshold δ | 0.7 |
| max epochs | 181 |
| the gate-loss weight $\alpha_{\mathrm{gate}}$ | 1 |
| the attention entropy weight $\alpha_{\mathrm{ent}}$ | 0.01 |
| balancing coefficient $\lambda$ | 0.4 |

**Table. S2. Summary of the preprocessed datasets.**

| **Datasets** | **WSIs** | **Spots** | **Genes** |
| --- | --- | --- | --- |
| HER2+ | 32 | 9612 | 785 |
| cSCC | 12 | 6630 | 171 |
| Alex | 6 | 25914 | 50 |

**References**

He,K. et al. (2016) Deep residual learning for image recognition. In: Proceedings of the IEEE Conference on Computer Vision and Pattern Recognition. pp. 770–778.

Johannet,P. et al. (2024) Molecular and clinicopathologic impact of gnas variants across solid tumors. J. Clin. Oncol., 42, 3847–3857.

Li,R.Q. et al. (2024) Cd74 as a prognostic and m1 macrophage infiltration marker in a comprehensive pan-cancer analysis. Sci. Rep., 14, 8125.

Menendez,J.A. and Lupu,R. (2017) Fatty acid synthase (fasn) as a therapeutic target in breast cancer. Expert Opin. Ther. Targets, 21, 1001–1016.

Murakami-Nishimagi,Y. et al. (2023) Claudin 4-adhesion signaling drives breast cancer metabolism and progression via liver X receptor β. Breast Cancer Res., 25, 41.

Noblejas-López,M.M. et al. (2019) Expression of mhc class i, hla-a and hla-b identifies immune-activated breast tumors with favorable outcome. Oncoimmunology, 8, e1629780.

Saraiva,D.P. et al. (2021) Expression of hla-dr in cytotoxic t lymphocytes: a validated predictive biomarker and a potential therapeutic strategy in breast cancer. Cancers, 13, 3841.

Sen,U. et al. (2023) Stearoyl coenzyme a desaturase-1: multitasker in cancer, metabolism, and ferroptosis. Trends Cancer, 9, 480–489.
